# Nutritional Status and Implementation of a Nutritional Education Program in Young Female Artistic Gymnasts

**DOI:** 10.3390/nu13051399

**Published:** 2021-04-21

**Authors:** Antoni Aguilo, Leticia Lozano, Pedro Tauler, Mar Nafría, Miquel Colom, Sonia Martínez

**Affiliations:** 1 Research Group on Evidence, Lifestyles and Health, Department of Nursing and Physiotherapy, Research Institute of Health Sciences (IUNICS), University of the Balearic Islands, 07122 Palma, Spain; aaguilo@uib.es (A.A.); leticia.lozano@uib.es (L.L.); marfnafria@gmail.com (M.N.); miquelcolomrossello@gmail.com (M.C.); sonia.martinez@uib.es (S.M.); 2 Health Research Institute of the Balearic Islands (IdISBa), 07120 Palma, Spain; 3 Research Group on Evidence, Lifestyles and Health, Department of Fundamental Biology and Health Sciences, Research Institute of Health Sciences (IUNICS), University of the Balearic Islands, 07122 Palma, Spain

**Keywords:** nutritional education, gymnasts, diet quality, nutritional knowledge, anthropometry

## Abstract

Adolescent high-performance gymnasts are considered to be at risk for low energy intake. The aim of the present study was to determine the effects of implementing a nutritional education program during the sports season on the nutritional status and nutrition knowledge of the female artistic gymnasts from the Technification Center of the Balearic Islands (n = 24; age, 14.1 ± 2.3 years). A quasi-experimental intervention design was applied, which consisted of implementing a nutritional education program of seven sessions given during eight months. Measurements of nutritional intake, nutrition knowledge, and anthropometric parameters, as well as hematological and biochemical blood parameters, were performed. Gymnasts reported low energy and carbohydrate intakes, with significant increases during the study (energy, 28.3 ± 1.4 vs. 32.8 ± 1.4 kcal kg^−1^, *p* = 0.015, carbohydrate 3.2 ± 0.2 vs. 3.9 ± 0.2 g kg^−1^, *p* = 0.004). The average values for parameters such as hemoglobin, ferritin, lipoprotein, and vitamin C and E levels in the plasma were within normal ranges. Low intakes of most of the food groups were observed during the study, with similar initial and final values. Nutrition knowledge did not change as a result of the study (28.0 ± 1.7 vs. 31.1 ± 1.3, *p* = 0.185). In conclusion, gymnasts reported low energy intakes. However, blood markers and most of the anthropometrical parameters measured were within normal ranges. The nutrition education program implemented did not produce significant improvements in the dietary habits or nutritional knowledge of gymnasts.

## 1. Introduction

Nutritional education is based on the transmission of information and use of tools related to food characteristics and nutritional content with the aim of improving dietary habits and, thus, health status [1]. Nutritional education is designed to assist and facilitate healthy eating choices and other healthy nutrition-related behaviors [2]. It has been shown that nutritional education improves the quality and variety of the diet in preadolescents and adolescents [3,4,5], as well as in athletes [5,6], leading to a healthy diet. Preadolescence and adolescence are key life periods in terms of biological growth and development. Therefore, during these stages, it is essential to cover all nutritional and energetic demands to allow proper development [7].

High-performance gymnasts are considered to be at risk for nutritional issues [8], with a higher risk of suffering from an eating disorder [9,10]. Several reasons have been proposed to explain this situation [11,12]. Among them is the aesthetic component of gymnastics and the requirement to maintain a low weight, leading to a decreased energy intake [13,14,15,16]. Furthermore, these athletes are usually engaged in very demanding training schedules with long sessions almost every day. This increases their energy demand, and they are under constant pressure to perform exercises with high precision and strength. Finally, gymnasts are mainly adolescents, or even pre-adolescents, periods that correspond to ages at which optimal performance can be achieved. Therefore, even during normal growth and development gymnasts still need to manage their stressful routines [7].

The relationship between having an adequate diet and proper performance in sport has been widely demonstrated. Among other requirements are optimal hydration, a good electrolyte balance, and adequate macro- and micronutrient intakes [17]. It has been suggested that an inadequate food intake combined with intense physical training in female elite gymnasts can, in combination with other factors such as the genetic potential or the hormonal environment, negatively influence the normal pattern of pubertal development (menstrual disorders, amenorrhea), as well as induce a delay in bone growth [18]. When high levels of physical activity are performed during adolescence, it is even more important to ensure an appropriate nutritional intake. In fact, it has been reported that in adolescent and preadolescent athletes, nutritional interventions become even more important [19].

The aim of the present study was to determine the effects of implementing a nutritional education program during the sports season on the nutritional status and knowledge of female artistic gymnasts from the Technification Center of the Balearic Islands. The initial nutritional and anthropometrical statuses of the gymnasts as well as changes in anthropometrical parameters during the study were analyzed.

## 2. Materials and Methods

### 2.1. Study Design and Participants

A quasi-experimental intervention design was applied. This consisted of the implementation of a nutritional education program for eight months in a group of young female artistic gymnasts. The protocol was developed in accordance with the Declaration of Helsinki for research of human participants and was approved by the Balearic Islands Clinical Investigation Ethics Committee (IB 1046/08 PI). All gymnasts, their parents, and their coaches were informed about the study. Participation was voluntary and participants or, in the case of minors, their parents, signed an informed consent prior to their inclusion.

Participants in the study were female gymnasts from the Sports Technification Center of the Balearic Islands (Palma, Mallorca, Spain). Initially, 25 participants were recruited, which included all gymnasts from the center, but one of them withdrew from the study because she left the sports center. Therefore, 24 gymnasts (average age, 14.1 ± 2.3 years) belonging to the following different formative levels completed the study: High-performance (n = 5), technification (n = 10), and follow-up (n = 9). All participants in the study were living with their parents at the family home. Gymnasts belonging to the high-performance group performed morning and afternoon training sessions. Gymnasts belonging to the technification and follow-up groups trained only in the afternoon and went to different schools. For all groups, the training sessions were held from Monday to Friday from 4:30 p.m. to 9 p.m. Additionally, on these days, the gymnasts from the high-performance group trained for 2–3 h in the morning. On Saturdays, a four-hour training session was performed (either in the morning or in the afternoon). This training routine was followed throughout the year except for a 15-day rest period during the summer holidays. The season was divided into four periods: Preparatory, precompetitive, competitive, and transitional.

### 2.2. Intervention

A nutritional education program was implemented from October 2008 to May 2009. This intervention started just before commencement of the more demanding training sessions, which aimed to improve endurance and resistance. The previous training sessions were mainly designed to increase flexibility and elasticity and promote basic technical concepts. This program consisted of seven formative sessions and activities for gymnasts and/or their parents. Additional nutritional information was given to the gymnasts in leaflets. Seven formative sessions were performed for durations of between 45 and 90 min once per month starting in the second month of the study. The contents of each formative session were based on the previous knowledge of the gymnasts. Briefly, the first session was about healthy eating and was addressed to the study group, coaches, and parents. In this session, the importance of good nutrition during the stages of development and its influence on the performance of high-performance physical activity were explained. The second session was about myths in food and was addressed to the group of gymnasts and their coaches. The third session was about hydration and was addressed to gymnasts, coaches, and parents. In addition, they were given information about hydration before, during, and after competition and training sessions by means of a written guide. In the fourth session, individualized advice was given after studying questionnaires carried out in previous months. The individual consultations lasted for about 15–30 min. Two of the sessions addressed the issue of healthy cooking for parents (Appendix A). The last session consisted of planning a healthy menu and was addressed to gymnasts, coaches, and parents. In the following months of study, the knowledge developed was reinforced, and information about preparation of mid-morning and afternoon snacks was provided.

### 2.3. Data Collection and Measurements

#### 2.3.1. Physical Activity Levels

The level of physical activity of the gymnasts; the intensity, assessed by the heart rate; and the energy expenditure during training sessions were measured using an electronic heart rate monitor (Suunto AMBIT3 PEAK). This methodology has been previously used in adolescents, showing an adequate accuracy for moderate to intense exercises [20,21,22].

#### 2.3.2. Dietary Habits and Nutrition Knowledge

Three 24-h recalls were completed at each of the following study time points: Beginning of the study, after three months, after six months, and at the end of the study. Macronutrient and micronutrient intakes were determined using the Food and Health analysis software version 9.0 (Nutrisalud; CSG Software, Huesca, Spain).

A semi-quantitative Food Frequency Questionnaire (FFQ) [23] was completed by the participants at the beginning and end of the study. The diversity of the diet (diet diversity score, DDS) and adherence to the Mediterranean diet were also determined at the beginning and end of the study. The DDS evaluates the daily consumption of foods from different food groups: (1) Protein-rich foods (meat, fish, and eggs); (2) calcium-rich foods (dairy products and legumes); (3) complex carbohydrate-rich foods (cereals and tubers); (4) fruits; and (5) vegetables. Two points were given to each individual consuming at least one serving of each food group, and 0 points were given when the level of consumption was below one serving. The DDS ranges from 0 to 10 points [24]. Adherence to the Mediterranean diet was determined using a 14-item questionnaire previously developed and validated for the Spanish population [25]. In this questionnaire, each item is scored as 0 or 1. A global score of 9 or higher indicates good adherence to the Mediterranean diet.

The gymnasts’ nutritional knowledge was measured before and after the nutritional education program using an adaptation of the validated general nutrition knowledge questionnaire [26]. This adaptation includes 61 items with each scored as 0 or 1.

The Eating Attitudes Test (EAT-26) was used at the beginning of the study to determine the presence of eating disorders. The 26-item self-reported test used was a validated short version of the EAT-40 that was presented in Spanish [27]. Six answers were offered for each question: Never, rarely, sometimes, often, usually, and always. Answers never, rarely, and sometimes were scored as “0”, often as “1”, usually as “2”, and always as “3”. The results of the test consisted of a total score obtained from three subscales: Dieting, bulimia and food preoccupation, and oral control. A global score of 20 or higher indicated an at-risk individual.

#### 2.3.3. Blood Sampling and Measurements

Blood samples were taken at the beginning and end of the study. Seated venous blood samples were collected in suitable vacutainers with ethylenediaminetetraacetic acid (EDTA) as an anticoagulant to obtain plasma and blood mononuclear cells (BMNC) and without additive to obtain serum. Plasma was obtained within 30 min after blood collection by centrifugation (15 min, 1000× *g*, 4 °C). Blood mononuclear cells were purified from an EDTA sample using Ficoll following the method described by Boyum [28]. Both plasma and BMNC samples were stored at −70 °C until measurements were performed. Serum was obtained by allowing the whole blood to clot for 30 min, followed by chilled centrifugation (15 min, 1000× *g*, 4 °C). Measurements were immediately performed in serum samples.

Hematocrit, hemoglobin, and blood cell counts were determined in an EDTA sample using a hematology analyzer (Horiba ABX Pentra 60, Diagnostics).

Vitamin C and vitamin E concentrations were measured in plasma and in blood mononuclear cells. Furthermore, retinol and carotene concentrations were determined in plasma. These measurements were performed using previously described high-performance liquid chromatography (HPLC) methods [29].

Concentrations of iron, ferritin, transferrin, total cholesterol, high density lipoprotein (HDL)-cholesterol, low density lipoprotein (LDL), triacylglycerides, glucose, urea, creatinine, uric acid, and bilirubin were measured in serum. Aspartate aminotransferase (AST), alanine aminotransferase (ALT), gamma-glutamyl transferase (GGT), and creatine kinase (CK) activities were also measured in serum. All serum parameters were determined following the standard procedures used in clinical biochemistry laboratories with an autoanalyzer. (SYNCHRON CXH9 PRO; Beckman Coulter, Brea, CA, USA).

#### 2.3.4. Anthropometric Parameters

Anthropometric measurements were conducted by well-trained researchers according to the recommendations of the International Standards for Anthropometric Assessment (ISAK) [30]. Body weight (electronic scale Seca 700; Seca, Hamburg, Germany) and height (Stadiometer Seca 220 CM Telescopic Height Rod for Column Scales, precision 0.5 cm; Seca) were measured, and body mass index was calculated as weight (kg) divided by height (m) squared.

Skinfolds (triceps, subscapular, abdominal, supraspinal, frontal thigh, and medial leg) were determined using a Harpender caliper (precision of 10 g/mm^2^). Fat mass, fat-free mass, and body water content were measured using a Body Composition Analyzer (Bioelectrical Impedance Analysis, Electromedicarin Bodycell model, multifrequency 1–150 kHz, Barcelona, Spain). To further characterize gymnasts, their somatotypes were determined at the beginning of the study following the Heath–Carter model [31].

#### 2.3.5. Statistical Analysis

The statistical analysis was carried out using the Statistical Package for Social Sciences (IBM SPSS Statistics 25.0 for Windows). The results were expressed as the means and standard deviations (SD), and *p* < 0.05 was considered statistically significant for all analyses. All data were assessed to determine whether a normal distribution was present (Shapiro–Wilk test). For parameters measured at the beginning and end of the study, the Student’s *t*-test for paired data was used to determine any significant difference between measures. When four measurements were performed for each subject, an ANOVA for repeated measures was applied.

## 3. Results

### 3.1. Dietary Intake, Quality of the Diet, and Nutrition Knowledge

Table 1 shows the changes in energy, macronutrient, and water intakes during the study. Significant changes were observed for energy, both when expressed as total energy (*p* = 0.002) and based on body weight (*p* = 0.0015), with values increasing at the fourth month of the study. However, energy intake was below the recommended level (Table S1) throughout the study. A significant increase in water intake was also observed towards the end of the study (*p* < 0.001). Carbohydrate consumption relative to body weight increased significantly (*p* = 0.004) because of increased simple (*p* = 0.021) and complex (*p* = 0.001) carbohydrate intakes. Furthermore, lipid intake increased (*p* = 0.006), leading to a decrease in the percentage of energy from proteins (*p* = 0.015). Regarding the consumption of fats, the intake of polyunsaturated fatty acids increased (*p* = 0.009), but there was no change in saturated and monounsaturated fatty acid intake.

Table 2 shows the changes in micronutrient intake during the study. Among the minerals, sodium intake increased (*p* = 0.004) while potassium (*p* = 0.007) and iron (*p* = 0.026) intakes decreased. Intake of vitamin A (*p* < 0.001), carotenoids (*p* < 0.001), and vitamin D (*p* < 0.001) decreased during the study. In contrast, vitamin E intake increased (*p* < 0.001). Significant changes were also observed for vitamin C intake, with higher initial values (*p* < 0.001). With regard to micronutrient requirements, calcium, zinc, vitamin E, and vitamin D intakes were below the average requirement. While initial vitamin A intakes were higher than recommended, a decrease was observed after the initial measurement, leading to more adequate intakes. Therefore, if hydrosoluble vitamins were not considered, only sodium intake at the end of the study and phosphorus intake throughout the study were above the recommended levels.

**Figure 1:**
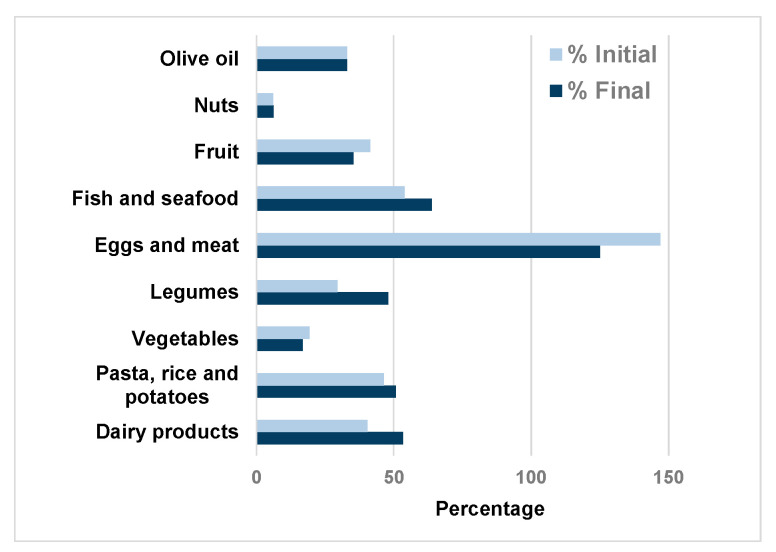
Intake of food groups with respect to recommendations at the beginning and end of the study. The results were expressed as the percentage of food group intake with respect to the recommendations for each group [32]. The 100% value represented the recommended intake.

Figure 1 shows the results of the FFQ at the beginning and end of the study. Intake levels of all food groups except for eggs and meat were below recommendations, both at the beginning and at the end of the study. Intake levels of eggs and meat were higher than the recommendations. No significant differences were found between the initial and final intakes.

Basal scores for adherence to the Mediterranean diet and DDS were 7.21 ± 0.35 and 2.26 ± 0.35, respectively. Both scores were maintained after the implementation of the nutritional education program (adherence to the Mediterranean diet: 7.56 ± 0.24, *p* = 0.119; DDS: 2.60 ± 0.17, *p* = 0.295). There was a slight increase in nutritional knowledge at the end of the study, 28.0 ± 1.7 vs. 31.1 ± 1.3 (*p* = 0.185). Regarding the initial EAT-26 test scores, the mean value was 10.8 ± 8.7, and only one out of the 24 gymnasts reported a score over 20 points.

### 3.2. Blood Cell Counts, Hematological Parameters, and Iron Metabolism

No significant differences in red blood cell related parameters or white blood cell counts were observed (Table 3). However, significant increases were observed in ferritin (*p* = 0.008) and transferrin (*p* < 0.001) concentrations. Two gymnasts presented initial hemoglobin values below 12 g/dl, and four gymnasts had ferritin concentrations below 15 ng/dl. At the end of the study period, all gymnasts were found to have adequate hemoglobin and ferritin values.

### 3.3. Biochemistry Parameters and Vitamin Levels

Table 4 shows the changes in serum biochemical parameters. Glucose levels decreased (*p* < 0.001) during the study. On the other hand, urea (*p* = 0.030), creatinine (*p* < 0.001), and uric acid (*p* < 0.001) serum concentrations increased significantly. Regarding the lipid profile, significant decreases in the total cholesterol (*p* < 0.001), LDL-cholesterol (*p* = 0.006), and HDL-cholesterol concentrations were observed (*p* < 0.001). No significant differences were found for serum enzyme activities.

The plasma vitamin E concentration decreased significantly (*p* = 0.012) during the study (Table 5). However, a significant increase in the vitamin A level was observed (*p* = 0.013). Concentrations of vitamins C and E in blood mononuclear cells did not change during the study. Regarding carotenes, a significant increase in the plasma cryptoxanthin level was observed (*p* = 0.003).

### 3.4. Physical Activity Levels and Anthropometrical Parameters

During the training sessions, the average heart rate recorded was 129 ± 2 beats·min^−1^, with a maximum value of 179 ± 2 beats·min^−1^. The average resting basal heart rate of the participants in the study was 74 ± 2 beats·min^−1^. The average energy expenditure during the training sessions was 878.5-± 38.7 kcal (21.54 ± 1.20 kcal·kg^−1^ body mass).

Gymnasts presented predominantly mesomorphic body types (2-6-3), with low values for the endomorphic component. Table 6 shows the changes in anthropometrical parameters. At the end of the study, significant increases in weight (*p* < 0.001), height (*p* < 0.001), BMI (*p* < 0.001), fat mass (*p* = 0.002), muscle mass (*p* < 0.001), and fat-free mass (*p* < 0.001) were observed. For skinfold measurements, only the lower leg measurement increased during the study (*p* < 0.001). The fat mass percentage decreased during the study (*p* = 0.003). However, the lean mass (*p* = 0.003) and water (*p* = 0.001) percentages as well as the total lean mass (*p* < 0.001) and water contents (*p* < 0.001) increased during the study.

## 4. Discussion

The main finding of the present study was the low energy intake of gymnasts. However, this low energy intake was not associated with impaired blood parameters or decreased intakes of most micronutrients. The nutritional intervention was not associated with significant changes in the main characteristics of the gymnasts’ diet.

In agreement with previous reports [13,14,15,16], energy intakes of gymnasts in the present study were below recommended levels for the age group and the level of physical activity performed [33]. This low energy intake was in agreement with the low intakes of almost all food groups reported in the FFQ. However, even with this low energy intake, gymnasts were not found to be at risk for developing eating disorders, with only one gymnast being identified as at risk. This result agrees with those obtained by Martínez-Rodríguez et al., who reported that only two out of 33 gymnasts of similar ages to the ones in the present study were at risk [34]. It is noteworthy that studies with female athletes from weight-class sports have been shown to have higher numbers of at-risk participants [35,36]. It is possible that sports involving practices to prevent increases in body weight, leading to individuals having to compete in a higher weight category, or strategies to lose weight to compete in a lower weight category, are more stressful than gymnastics, in which aesthetics is the main reason for maintaining a low weight.

While the percentage of energy from carbohydrates was within the recommended range, in agreement with previous results [37], the low energy intake was associated with a low total intake of carbohydrates. According to specific recommendations for athletes, an intake of up to 5–6 g carbohydrate/kg is necessary, taking into account that the gymnasts follow a very demanding training schedule [38]. Although carbohydrate increased from 3.2 ± 0.2 to 3.9 ± 0.2 g kg^−1^ by the end of the study, this was still not enough to attain recommended levels for these athletes. While a previous study conducted in university athletes reported an increase in carbohydrate intake after a nutritional intervention [39], in the present study, the reason for this increase may have been it being conducted at the beginning of the more demanding training season, rather than the nutritional intervention. In addition to being low, the carbohydrate intake was characterized by a high proportion of simple carbohydrates. Despite the different nutritional characteristics of simple and complex carbohydrates being established in the nutritional education program, the deviation in the proportion of complex to simple carbohydrates was not corrected by the end of the study. Considering the results of the food group intakes, the sources of this high simple carbohydrate intake were not clear. In this regard, biases due to under or misreporting of, mainly, unhealthy foods are commonly found when dietary records are performed [40,41]. Further, pressure from parents and/or coaches can lead to bias in the information provided by the athletes themselves [42].

The increase in carbohydrate intake contributed to the higher total energy intake observed three months after the beginning of the study, a result also found when energy intake was expressed relative to body weight. It is noteworthy that changes observed in the total energy intake were produced within the initial three months of the study, without additional modifications until the end of the program. This increased energy intake was not associated with the increase in body weight observed at the end of the study. The percentage of energy from fat was within the recommended range both at the beginning and at the end of the study, which is in agreement with previous results [37,43]. However, it should be considered that fat intake also increased during the initial study period, mainly due to an increase in unsaturated fat intake. In addition, the percentage of energy produced from proteins decreased, but values remained within the recommended range throughout the study [44]. This change in the percentage of energy intake from proteins could be attributed to the increases in carbohydrate and lipid intakes, rather than being due to a decrease in protein intake per se. In spite of the higher intake than that recommended for the general population, the protein intake 1.6–1.7 g·kg^−1^ body mass could be considered adequate, because daily intakes of between 1.2 and 2.0 g·kg^−1^ have been suggested for athletes [17]. The high proportion of animal to vegetable protein intake observed in the present study could reflect high in meat and egg intakes and low legume and nut intakes. The high phosphorus intake could be also related to the high intakes of foods such as meat and eggs.

Dietary iron is an essential nutrient for human health and for maintaining athletes’ performance [45]. Low levels of iron have commonly been found, above all, in female, mainly young, athletes with low caloric or iron intakes, and consequently, these athletes have hematological indices below the reference values [46,47]. In the present study, and in spite of the decrease observed after the first measurements were taken, iron intake was slightly above the recommended values. Because it has been suggested that the iron intakes of female athletes should be increased by 70% from the estimated average requirement [17], higher intakes in this group of gymnasts could be deemed adequate. Actually, periods of rapid growth, such as the one being experienced by these gymnasts, can negatively impact iron status [17]. However, average plasma and blood values related to iron metabolism, namely, hemoglobin and ferritin, were within the normal range, and there were even increased levels of ferritin and transferrin at the end of the study. In fact, as indicated above, at the end of the study period, all participants presented values of the main markers hemoglobin and ferritin that were within the healthy range. Similar results have been obtained in previous studies involving gymnasts [16,48], suggesting that gymnasts do not commonly present alterations in iron metabolism, and there is a low incidence of depletion in iron stores [16,48,49]. However, others have reported that poor iron status is commonly found in female athletes in general [46] and in young female athletes in particular [46]. In the present study, it is possible that the high intakes of eggs and meat, together with the beneficious interaction between iron and vitamin C intakes [17], induced a healthier iron status than that found in previous studies [46,48].

In addition to the adequate levels of blood iron metabolism related parameters, vitamin levels analyzed in the blood were also within the normal range [50]. While both vitamin A and vitamin C blood levels reflected adequate intakes, it is striking that vitamin E levels were within the normal range despite intake being below recommended values. In this regard, a poor correlation between intake and plasma levels of vitamin E has been reported because vitamin E is mainly transported by lipoproteins, and, therefore, levels of these lipoproteins could determine plasma vitamin E levels [51]. In this sense, the decrease in plasma vitamin E observed at the end of the study could have been induced by the decreases in lipoprotein (LDL and HDL) levels observed. The lack of significant changes in the vitamin E concentration in blood mononuclear cells could support the idea that the lipoprotein levels are important determinants of the plasma concentration and could indicate a proper vitamin E status in gymnasts. With regard to the lipoprotein levels in gymnasts, only slightly elevated initial LDL-cholesterol levels were observed, which improved after the aforementioned decrease, leading to values within the recommended range. This decrease in LDL-cholesterol levels is in agreement with the results of a previous nutritional intervention in university athletes [39]. However, this decrease could have been also induced by the training season, as it has been suggested that in children and adolescents, decreases in LDL-cholesterol are associated with an increased training intensity [52]. On the other hand, cholesterol levels at the end of the study were similar to the ones found by Guerra et al. [43]. Therefore, the presence of an adequate lipid intake is in agreement with the results of the serum markers analyzed as well as with the healthy values found for the determined cholesterol ratios, which showed an adequate lipid profile with no cardiovascular risk [53].

There is an emerging interest in the biomolecular roles of vitamin D in general and in athletes in particular [17]. In agreement with previous studies conducted on elite gymnasts [18,54], the vitamin D intake of participants in the present study was lower than recommended. The low intake of vitamin D could have been associated with the low calcium intake. The low values for calcium and vitamin D could reflect the low intake of dairy products recorded in these athletes. Because plasma levels of vitamin D were not measured, it could not be ascertained as to whether this low intake could induce a deficit. These athletes do not live at latitudes over the thirty-fifth parallel, which has been suggested as being a negative factor in attaining optimal vitamin D levels. However, they primarily train and compete indoors, which could lead to insufficient ultraviolet B exposure. More studies are needed to clarify whether gymnasts are at risk for vitamin D deficit as well as the possible consequences of this deficit.

Previous studies have reported that nutritional education programs induced improvements not only in the nutritional knowledge of athletes [55,56], but also in introducing more positive dietary changes [5,6]. In the present study, the lack of significant improvements in the gymnasts’ dietary habits could be related to the slight non-significant increase observed in the nutrition knowledge scores. In this regard, it has been suggested that nutritional education can assist and facilitate healthy eating choices as well as healthy nutrition-related behaviors [2]. However, in the present study, the nutritional education program did not induce a significant improvement in the gymnasts’ nutritional knowledge. This was within individuals with a low level of general nutritional knowledge, which did not reach half of the maximum range score. While nutritional knowledge did not increase, we were unlikely to find significant changes leading to healthy choices. In fact, and in addition to observations indicated above, such as the high intakes of simple carbohydrates and animal protein, the low adherence to the Mediterranean diet indicated low-quality dietary habits of the gymnasts, which did not improve during the study. This result is in agreement with the observation of worse healthy habits in adolescents compared with the general population [57].

It has been suggested that somatotypes strongly influence sports performance in general and in gymnasts in particular [58]. Previous studies have shown that gymnasts predominantly have a mesomorphic somatotype (2-6-3), with low values of the endomorphic component [59,60], which is in agreement with results from the present study. Despite gymnasts being, at the beginning of the study, in percentiles slightly below the average values [61], ranging from 10 to 25 (results not shown), they reported an adequate weight in relation to height. At the end of the study, they attained percentiles within the normal range, close to P50, with a significant increase in body weight. This weight increase could be attributed to the increases in height and fat-free mass without increases in fat mass. All of these results are consistent with the body compositions reflected in other studies [60,62,63] and correlate with the evaluated somatotypes [63]. In this regard, similar results to those obtained in the present study regarding BMI, fat percentage, and fat-free mass have been shown previously [9,36,37]. The low fat mass percentage commonly observed in gymnasts has not been associated with health issues [63]. In fact, it has been reported that anthropometrical changes observed in gymnasts during growth present a similar pattern to that of the non-athlete female population [64]. In this regard, it was also suggested that the negative balance between caloric intake and energy expenditure represents short-term data and does not seem to affect normal growth [65]. Similarly, a previous study performed in young gymnasts and swimmers found no evidence of a negative influence of demanding physical activity from an early age on growth until puberty [66], and energy deficit has not been commonly associated with slowing down of growth and maturation in gymnasts [65]. This is in spite of other authors arguing that inadequate food intake combined with exercise can alter the normal pattern of growth and pubertal development [18].

The present study presented some limitations that should be acknowledged. The main limitation was the study design. We used a “quasi-experimental design” because of the impossibility of finding another group of participants with similar characteristics. Participants in the study were the only group with the characteristics described in Mallorca. Furthermore, the implementation of the nutrition education program to half of the sample, when the benefits of these programs have been supported by previous studies, was considered ethically inappropriate. In addition, a high degree of contamination between groups could be expected. Repetitive measures (pre- and post-intervention, as well as two intermediate measurements) were taken, with each subject being its own control to prevent, in part, limitations derived from the lack of a control group. The small sample size was also a major limitation, and all conclusions of the study were based on this small sample size and may not apply to other subjects. Regarding the methods applied, the use of three 24-h recalls to determine micronutrient intake did not allow proper measurement. In this regard, and as has been indicated above, when dietary records were performed in populations such as that used in the present study, under and miss-reporting biases could be produced [40,41]. However, the measurement of blood, plasma, and cell parameters was a more confident way to analyze the micronutrient status of gymnasts.

## 5. Conclusions

Gymnasts were found to have low energy intakes, mainly due to having insufficient carbohydrate intakes and generally low intakes of most food groups. In spite of these observations, blood markers and most anthropometrical parameters measured were within adequate ranges. However, the nutrition education program implemented did not produce significant improvements in dietary habits or in the nutritional knowledge of gymnasts. It is possible that the nutritional intervention applied was not adequate due to the contents considered, the frequency and number of formative sessions, or the short duration of the whole intervention.

## Figures and Tables

**Table 1:** Changes in daily energy, macronutrient, and water intakes during the study.

	Initial	Three Months	Six Months	Final	*p*-Value
**Energy (kcal)**	1172 ± 57	1415 ± 42	1366 ± 53	1454 ± 60	0.002 *
**Energy (kcal·kg^−1^)**	28.3 ± 1.4	33.5 ±1.0	31.3 ± 1.2	32.8 ± 1.4	0.015 *
**Water (mL)**	1636 ± 74	1590 ± 62	1846 ± 75	1989 ± 80	<0.001 *
**CHO (%)**	45.3 ± 1.6	49.6 ± 1.2	48.4 ± 1.4	47.6 ± 1.5	0.167
**CHO (g·kg^−1^)**	3.2 ± 0.2	4.1 ± 0.1	3.8 ± 0.2	3.9 ± 0.2	0.004 *
**Simple CHO (g·kg^−1^)**	1.7 ± 0.1	1.9 ± 0.7	1.8 ± 0.1	2.2 ± 0.2	0.021 *
**Complex CHO (g·kg^−1^)**	1.5 ± 0.1	2.2 ± 0.1	2.0 ± 0.1	1.7 ± 0.1	0.001 *
**Fiber (g)**	14.6 ± 1.1	12.5 ± 0.8	12.3 ± 0.8	12.7 ± 0.81	0.237
**Protein (%)**	23.6 ± 1.3	20.5 ± 0.6	20.4 ± 0.7	19.9 ± 0.8	0.015 *
**Protein (g·kg^−1^)**	1.6 ± 0.1	1.7 ± 0.1	1.6 ± 0.1	1.6 ± 0.1	0.417
**Animal protein (g·kg^−1^)**	1.2 ± 0.1	1.3 ± 0.1	1.1 ± 0.1	1.1 ± 0.1	0.414
**Vegetal protein (g·kg^−1^)**	0.5 ± 0.1	0.4 ± 0.1	0.4 ± 0.1	0.4 ± 0.1	0.611
**Lipids (%)**	31.1 ± 1.4	29.9 ± 1.0	31.2 ± 1.3	32.7 ± 1.3	0.431
**Lipids (g·kg^−1^)**	1.0 ± 0.1	1.1 ± 0.1	1.1 ± 0.1	1.2 ± 0.1	0.006 *
**SFA (g·kg^−1^)**	0.4 ± 0.1	0.5 ± 0.1	0.4 ± 0.1	0.43 ± 0.1	0.346
**MUFA (g·kg^−1^)**	0.4 ± 0.1	0.5 ± 0.1	0.5 ± 0.1	0.54 ± 0.1	0.082
**PUFA (g·kg^−1^)**	0.2 ± 0.1	0.2 ± 0.1	0.2 ± 0.1	0.22 ± 0.1	0.009 *
**Cholesterol (mg)**	244.2 ± 22.1	241.6 ± 18.5	218.0 ± 16.7	234.2 ± 20.4	0.798

* Indicates statistically significant differences within the season (*p* < 0.05). CHO: Carbohydrates; SFA: Saturated fatty acids; MUFA: Monounsaturated fatty acids; PUFA: Polyunsaturated fatty acids.

**Table 2:** Changes in daily energy, macronutrient, and water intakes during the study.

	Initial	Three Months	Six Months	Final	*p*-Value
**Sodium (mg)**	1844 ± 168	2292 ± 134	2590 ± 165	2614 ± 168	0.004 *
**Potassium (mg)**	2796 ± 154	2339 ± 89	2316 ± 110	2314 ± 91	0.007 *
**Calcium (mg)**	708.8 ± 36.7	662.5 ± 24.1	712.5 ± 32.2	705.8 ± 38.3	0.592
**Magnesium (mg)**	228.6 ± 11.2	224.4 ± 7.0	244.7 ± 14.3	241.6 ± 8.5	0.397
**Phosphorus (mg)**	996.3 ± 42.6	1022 ± 32	1022 ± 42	1051 ± 43	0.828
**Iron (mg)**	10.1 ± 0.6	8.4 ± 0.3	8.5 ± 0.4	8.7 ± 0.4	0.026 *
**Zinc (mg)**	7.1 ± 0.5	6.7 ± 0.3	6.9 ± 0.3	7.1 ± 0.3	0.833
**Vitamin A (µg)**	954.3 ± 115.6	517.0 ± 61.5	564.7 ± 81.6	576.6 ± 72.4	0.001 *
**Carotenoids (µg)**	4604 ± 684	1886 ± 332	2109 ± 420	2433 ± 400	<0.001 *
**Vitamin D (µg)**	2.1 ± 0.3	1.0 ± 0.1	1.1 ± 0.2	1.4 ± 0.2	<0.001 *
**Vitamin E (mg)**	6.5 ± 0.4	4.7 ± 0.3	4.8 ± 0.3	6.6 ± 0.5	<0.001 *
**Vitamin B1(mg)**	1.5 ± 0.2	1.1 ± 0.1	1.2 ± 0.1	1.9 ± 0.2	0.002 *
**Vitamin B2(mg)**	1.6 ± 0.2	1.4 ± 0.1	1.5 ± 0.1	1.7 ± 0.1	0.100
**Vitamin B3(mg)**	22.1± 1.7	17.4 ± 0.9	17.0 ± 1.2	17.1± 1.1	0.015 *
**Vitamin B6 (mg)**	1.9 ± 0.1	1.6 ± 0.1	1.6 ± 0.1	1.6 ± 0.1	0.109
**Vitamin B9 (µg)**	235.4 ± 16.7	193.4 ± 11.4	211.0 ± 16.7	208.9 ± 14.3	0.214
**Vitamin B12 (µg)**	4.8 ± 0.7	3.1 ± 0.2	3.1 ± 0.4	3.0 ± 0.2	0.004 *
**Vitamin C (mg)**	139.0 ± 15.7	77.1 ± 7.3	88.2 ± 10.7	102.0 ± 9.7	<0.001 *

* Indicates statistically significant differences within the season (*p* < 0.05). CHO: Carbohydrates; SFA: Saturated fatty acids; MUFA: Monounsaturated fatty acids; PUFA: Polyunsaturated fatty acids.

**Table 3:** Changes in blood cell counts, hematological parameters, and iron metabolism during the study.

	Initial	Final	*p*-Value
**Erythrocytes (10^6^·µL^−1^)**	4.72 ± 0.10	4.67 ± 0.10	0.226
**Hemoglobin (g·dL^−1^)**	13.3 ± 0.2	13.4 ± 0.1	0.116
**Hematocrit (%)**	40.3 ± 0.5	39.5 ± 0.4	0.029
**Platelets (10^3^·µL^−1^)**	288.3 ± 13.0	290.0 ± 12.7	0.833
**Leukocytes (10^3^·µL^−1^)**	6.02 ± 0.23	5.91 ± 0.22	0.621
**Neutrophils (10^3^·µL^−1^)**	3.00 ± 0.24	2.86 ± 0.12	0.534
**Lymphocytes (10^3^·µL^−1^)**	2.20 ± 0.13	2.28 ± 0.11	0.417
**Iron (µg·dL^−1^)**	79.6 ± 7.3	89.5 ± 7.8	0.282
**Ferritin (ng·dL^−1^)**	26.6 ± 4.3	39.6 ± 3.8	0.008 *
**Transferrin (mg·dL^−1^)**	240.5 ± 5.3	281.9 ± 8.3	<0.001 *
**Saturation Index (%)**	23.6 ± 2.1	25.5 ± 2.4	0.460

* Indicates statistically significant differences (initial vs. final; *p* < 0.05).

**Table 4:** Changes in serum biochemical parameters during the study.

	Initial	Final	*p*-Value
**Glucose (mg·dL^−1^)**	98.3 ± 2.0	90.5 ± 1.0	<0.001 *
**Urea (g·L^−1^)**	0.27 ± 0.01	0.31 ± 0.01	0.030 *
**Creatinine (mg·dL^−1^)**	0.69 ± 0.01	0.76 ± 0.02	<0.001 *
**Uric acid (mg·dL^−1^)**	3.70 ± 0.09	4.14 ± 0.15	<0.001 *
**Bilirubin (mg·dL^−1^)**	0.81 ± 0.08	0.70 ± 0.06	0.239
**Total cholesterol (mg·dL^−1^)**	179.9 ± 5.2	162.6 ± 5.1	<0.001 *
**HDL-cholesterol (mg·dL^−1^)**	61.6 ± 2.9	57.1 ± 2.5	0.006 *
**LDL-cholesterol (mg·dL^−1^)**	110.2 ± 4.5	97.8 ± 3.9	<0.001 *
**Cholesterol to HDL ratio**	3.00 ± 0.47	2.91 ± 0.46	0.103
**LDL-chol to HDL-chol ratio**	1.86 ± 0.45	1.77 ± 0.41	0.076
**Triglycerides (mg·dL^−1^)**	40.3 ± 2.2	38.5 ± 3.0	0.524
**AST (U·L^−1^)**	27.6 ± 1.1	27.8 ± 0.9	0.802
**ALT (U·L^−1^)**	17.9 ± 1.0	18.7 ± 0.6	0.429
**GGT (U·L^−1^)**	10.5 ± 0.3	10.6 ± 0.4	0.695
**CK (U·L^−1^)**	251.9 ± 26.9	294.3 ± 60.9	0.369

* Indicates statistically significant differences after vs. before (*p* < 0.05). HDL: High-density lipoprotein; LDL: Low-density lipoprotein; cholesterol-to-HDL ratio indicates the ratio of total cholesterol to HDL cholesterol; LDL-chol-to-HDL-chol ratio indicates the ratio of LDL-cholesterol to HDL-cholesterol; AST: Aspartate aminotransaminase; ALT: Alanine aminotransferase; GGT: Gamma-glutamyl transferase; CK: Creatine kinase.

**Table 5:** Changes in vitamin and carotene concentrations in plasma and blood mononuclear cells.

	Initial	Final	*p*-Value
**Plasma**			
**Vitamin E (µg·mL^−1^)**	8.10 ± 0.27	7.59 ± 0.24	0.012 *
**Vitamin A (µg·L^−1^)**	387.6 ± 13.6	414.8 ± 13.6	0.013 *
**Vitamin C (µg·mL^−1^)**	16.5 ± 0.9	15.3 ± 0.9	0.185
**Lutein (µg·L^−1^)**	109.2 ± 4.2	111.0 ± 3.7	0.657
**Cryptoxanthin (µg·l^−1^)**	65.7 ± 8.4	83.7± 10.0	0.003 *
**Lycopene (µg·L^−1^)**	134.4 ± 10.2	119.5 ± 8.9	0.148
**Carotene (µg·L^−1^)**	354.4 ± 30.1	317.8 ± 28.9	0.239
**Blood mononuclear cells**			
**Vitamin E (mM)**	1.16 ± 0.10	1.06 ± 0.07	0.263
**Vitamin C (mM)**	4.20 ± 0.27	3.96 ± 0.20	0.232

* Indicates statistically significant differences within the season (*p* < 0.05).

**Table 6:** Changes in gymnasts’ anthropometric parameters during the study.

	Initial	Three Months	Six Months	Final	*p*-Value
**Weight (kg)**	41.3 ± 9.9	42.0 ± 8.7	43.7 ± 8.7	44.2 ± 8.4	<0.001 *
**Height (cm)**	148.4 ± 9.7	148.7 ± 9.6	150.6 ± 9.1	151.0 ± 8.9	<0.001 *
**BMI (kg·m^−2^)**	18.6 ± 2.0	18.9 ± 2.0	19.0 ± 2.0	19.2 ± 2.0	<0.001 *
**Triceps skinfold (mm)**	6.9 ± 1.9	7.3 ± 1.9	6.6 ± 1.4	7.0 ± 1.7	0.404
**Subscapular skinfold (mm)**	5.5 ± 1.4	5.5± 1.4	5.6 ± 1.6	5.7 ± 1.5	0.102
**Suprailiac skinfold (mm)**	5.9 ±1.7	6.1 ±2.0	5.3 ± 1.6	5.2 ± 1.5	0.005 *
**Abdominal skinfold**	4.9 ± 1.6	5.3 ± 1.8	5.4 ± 1.8	5.6 ± 2.1	0.053
**Thigh skinfold**	12.4 ± 3.7	11.2 ± 2.9	12.1 ± 3.4	12.1 ± 3.7	0.932
**Lower leg skinfold (mm)**	9.7 ± 2.4	9.7 ± 1.9	10.3 ± 2.2	11.0 ± 2.3	<0.001 *
**Fat mass (%)**	17.7 ± 8.7	15.8 ± 6.8	15.3 ± 6.2	15.2 ± 6.8	0.003 *
**Fat mass (kg)**	7.92 ± 5.21	8.71 ± 8.23	7.06 ± 4.00	7.07 ± 4.13	0.459
**Fat free mass (%)**	81.0 ± 9.0	84.2 ± 6.8	84.7 ± 6.2	84.8 ± 6.8	0.003 *
**Fat free mass (kg)**	33.0 ± 5.0	35.2 ± 5.1	36.5 ± 5.3	36.9 ± 5.2	<0.001 *
**Water content (%)**	56.8 ± 5.4	60.2 ± 6.1	60.4 ± 4.0	60.1 ± 3.9	0.001 *
**Water content (kg)**	23.6 ± 3.8	24.9 ± 4.0	25.9 ± 4.2	26.2 ± 4.1	<0.001 *

BMI: Body mass index; * indicates statistically significant differences within the season (*p* < 0.05).

## Data Availability

The data presented in this study are available on request from the corresponding author. The data are not publicly available due to availability restrictions reported in the informed consent signed by parents of all participants.

## Supplementary Materials

The following are available online at https://www.mdpi.com/article/10.3390/nu13051399/s1.

## Appendix A

Workshop on Healthy Cooking (Main Contents)

1. The meaning of a healthy and balanced diet in athletes, demystifying errors such as:
  - “They must consume a lot of pasta and chicken”, explaining that consumption that is not varied and at the same time unbalanced leads to monotony and poor nutrition;
  - “Carbohydrates make you fat”, explained how excessive consumption leads to low performance and ketosis production occurs if they are eliminated from the diet;
  - “Little oil because it fattens”, leads to a low palatable and unbalanced diet;
  - “Bananas make you fatter than other fruits”;
  - “The egg is a very nutritious food”;
  - “Drinking water between meals makes you fat”, this myth leads to low water consumption, but this premise is false;
2. The functions and food sources of proteins (animal and vegetable origin), lipids (saturated and unsaturated), carbohydrates (simple and complex), minerals, vitamins, and fiber;
3. The food pyramid, emphasizing the adequate frequency of consumption of different food groups and the importance of daily intakes of nutrients;
4. The composition of a balanced breakfast and the necessity of breakfast. Examples;
5. The compositions of snacks, both mid-morning and afternoon. Examples;
6. Composition of meals and dinners need to be balanced (first course, second course, and dessert or combined dish and dessert). Examples. Complement meals with dinners, examples of weekly menus;
7. Differences between whole and refined foods;
8. Introduction to “new foods”, that is, foods that are not typical of the area or were not usually consumed and are nutritious foods. The methods of preparation of these foods were explained and tastings were performed. Among the foods explained were quinoa, buckwheat, amaranth, millet, rooibos, tofu, seitan, and tempeh;
9. Recipe for a homemade isotonic drink;
10. Resolution of any doubts and questions.
