# Supplementary material for: Nutritional Status and Implementation of a Nutritional Education Program in Young Female Artistic Gymnasts
Source: Nutrients. 2021 Apr 21;13(5):1399. doi: 10.3390/nu13051399 (PMC8143314; doi:10.3390/nu13051399)
Supplement: Supplementary file 1 [file nutrients-13-01399-s001.zip › nutrients-1183681-supplementary.pdf]

nutrients

MDPI

Table S1. Nutrient recommendations considered (European Food Safety Authority [33]).

Parameter	Recommendations	Parameter	Recommendations
**Energy (kcal)**	2447–2719	**Magnesium (mg)**	250
**Water (mL)**	1900	**Phosphorus (mg)**	640
**CHO (%)**	45–60	**Iron (mg)**	7–13 #
**Fiber (g)**	19	**Zinc (mg)**	8.9
**Protein (g·kg^{-1})**	0.87 #	**Vitamin A (μg)**	480–600 μg #
**Lipids (%)**	20–35	**Vitamin D (μg)**	15
**Fiber (g)**	19	**Vitamin E (mg)**	11
**SFA (g·kg^{-1})**	As low as possible	**Vitamin B1(mg)**	0.73
**Sodium (mg)**	2000	**Vitamin B2(mg)**	1.1
**Potassium (mg)**	2700	**Vitamin B3(mg)**	13.3
**Calcium (mg)**	960	**Vitamin B6 (mg)**	0.73
**Magnesium (mg)**	250	**Vitamin B9 (μg)**	210 μg
**Phosphorus (mg)**	640	**Vitamin B12 (μg)**	3.5
**Iron (mg)**	7–13 #	**Vitamin C (mg)**	60–70 #
**Zinc (mg)**	8.9		

SFA: Saturated fatty acids; unless stated, the European Food Safety Authority (EFSA) average requirement (AR) recommendations for general population adjusted per sex and age were used; energy intake considering physical activity (PAL) of 1.8 (active) to 2.0 (very active lifestyles) is stated; following the suggestions of the EFSA, vitamin B1 and B3 AR were adjusted to gymnasts' energy intakes; # indicates that the population reference intake (PRI) was used for that nutrient.
